# Gingival Vitiligo: Report of a Case and Review of the Literature

**DOI:** 10.1155/2014/874025

**Published:** 2014-06-11

**Authors:** Nipun Ashok, Anila Karunakaran, Prabath Singh, Jean Rodrigues, Navya Ashok, Bassel Tarakji, Azzeghaiby Saleh, Alzoghaibi Ibrahim

**Affiliations:** ^1^Department of Periodontics, Al Farabi College of Dentistry, Riyadh 11691, Saudi Arabia; ^2^Department of Oral Pathology, Kannur Dental College, Kannur 670612, India; ^3^Department of Endodontics, Amrita Institute of Dental Sciences, Cochin 682041, India; ^4^Department of Endodontics, Al Farabi College of Dentistry, Riyadh 11691, Saudi Arabia; ^5^Department of Orthodontics, Amrita Institute of Dental Sciences, Cochin 682041, India; ^6^Department of Oral and Maxillofacial Sciences, Al Farabi College of Dentistry, Riyadh 11691, Saudi Arabia; ^7^Al Farabi College of Dentistry, Riyadh 11691, Saudi Arabia

## Abstract

Rarely cases have been reported regarding depigmented lesions of the oral cavity. On reviewing the literature, only few cases of gingival vitiligo or similar lesions have been reported till date. These lesions pose a cosmetic challenge. We present here a case of vitiligo affecting gingiva. Vitiligo has been defined as an acquired, slowly progressive loss of cutaneous pigment which occurs as irregular, sharply defined patches which may or may not be surrounded by macroscopic hyperpigmentation. Differential diagnosis, detailed clinical history, histopathology, immunohistochemistry, and pathogenesis of this condition are discussed.

## 1. Introduction

Colour changes in skin and mucosa are of diagnostic importance and may present only as a mere cosmetic disfigurement or may indicate an underlying systemic disease. Gingival pigmentation is a rarity in Caucasian population but is commonly observed in Africans, East Asians, and Hispanics [1].

After reviewing the literature, it was observed that only very few cases of depigmented lesions have been reported in the oral mucosa. This depigmentation on skin or mucosa is termed as “vitiligo” or “leukopathia” or “piebaldism” or “leukoderma” [2]. Vitiligo has been defined as an acquired, slowly progressive loss of cutaneous pigment which occurs as irregular, sharply defined patches which may or may not be surrounded by macroscopic hyperpigmentation [3].

Gingival pigmentation plays an important role in the overall gingival colour and aesthetics. Vitiligo of gingiva could result in cosmetic debility. This case of gingival vitiligo is reported on account of its rarity and the cosmetic challenges it poses.

## 2. Prevalence

Vitiligo affects approximately 0.1–4% of the general population and has no racial or regional predilection. A positive family history has been observed in vitiligo cases suggestive of a genetic involvement. Females are more commonly affected [4].

## 3. Case Report

A 20-year-old female patient reported to the Department of Periodontics, Kannur Dental College, Kerala, with a chief complaint of a white patch on her gums which was present for the past 2 years. The lesion was asymptomatic and increased in size gradually during the course of the first year and has remained stable since then. The patient was otherwise healthy and was not under any medication. There was no history of alcohol consumption, smoking, and areca nut chewing habits. Neither was there a familial history of similar disease nor a history of trauma in the concerned site. The patient was concerned about the sudden change in the colour of her gingiva and reported to the hospital to find out the underlying cause.

On physical examination, the patient was moderately built and nourished and had a moderate brown skin colour (TYPE IV according to Fitzpatrick scale) [5]. Extra oral examination revealed no abnormal findings. There was no abnormality present anywhere else on the body.

**Figure 1:**
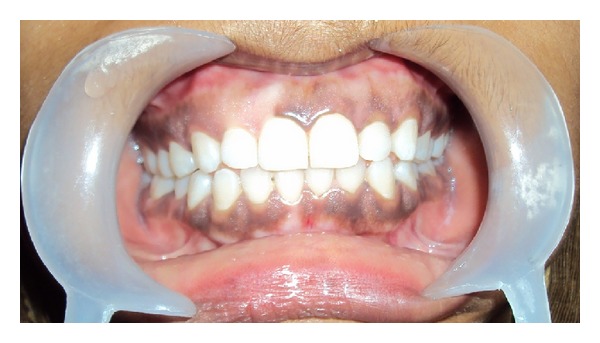
Clinical presentation shows white patch on the gingiva.

Intraorally, in the maxillary arch, there was a diffused white patch of 1.5 cm × 1 cm involving the gingiva extending from the middle of the right central incisor (8) to the distal aspect of canine (6) posteriorly, from the attached gingiva to the mucogingival junction (Figure 1). A similar diffused white patch of 1 × 0.5 cm was present on the gingiva in the left side of the maxilla extending from the distal half of the central incisor (9) to the canine region (11) but was less pronounced compared to the right side.

On inspection of gingiva in the mandible, a similar white patch of 0.5 cm × 0.5 cm was observed in the attached gingiva of the mandibular right central incisor (25). There was no other abnormality detected intraorally.

## 4. Histopathology

**Figure 2:**
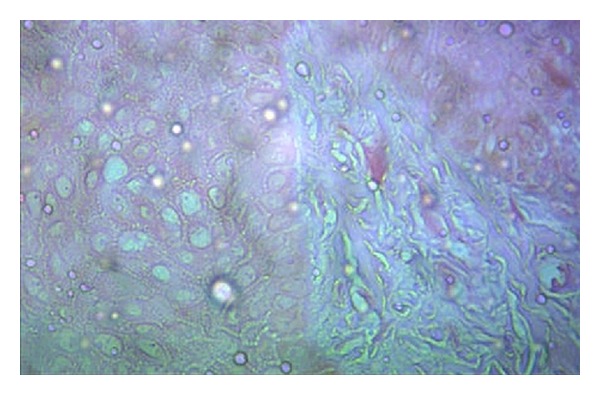
Photomicrograph of epithelium with vitiligo which is devoid of melanocytes (hematoxylin-eosin).

**Figure 3:**
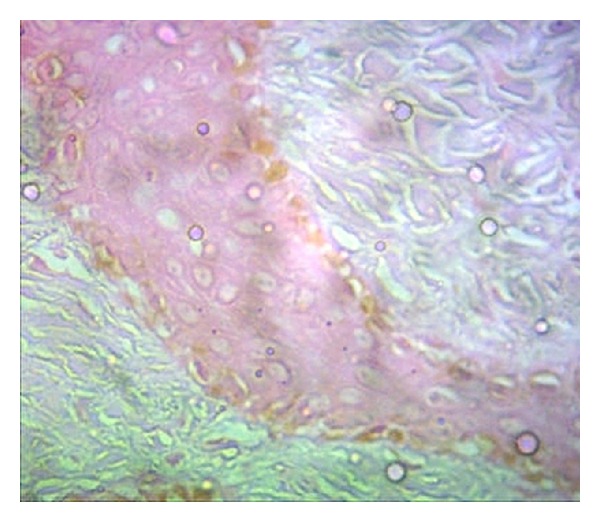
Photomicrograph of normal epithelium with melanocytes (hematoxylin-eosin).

**Figure 4:**
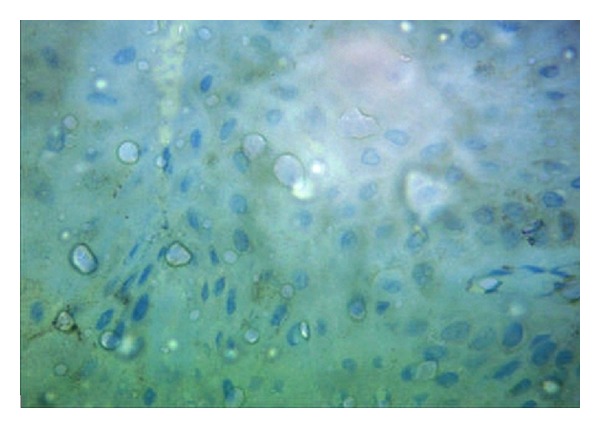
Photomicrograph showing absence of immunoreactivity for HMB-45 in gingival vitiligo.

An incisional biopsy was obtained involving the white patch and the surrounding normal tissue from the maxillary central incisor region. The microscopic examination of the section stained with hematoxylin and eosin showed very mild or no pigmentation in the epithelium (Figure 2) when compared to the tissue taken from the adjacent normal area (Figure 3). Epidermal vacuolization was noted and the underlying connective tissue was loosely fibrous with no evidence of inflammatory cells. Melanocytes present in the depigmented area did not show immunoreactivity for HMB-45 antibody which confirms the presence of vitiligo (Figure 4).

## 5. Discussion

Vitiligo affecting the skin has been commonly reported [6]. Most common sites of involvement are neck, face, and scalp. Mucosal involvement is seen in lips, genitals, gingiva, areola, and nipples [7, 8]. Incidence of vitiligo in oral tissues is rare. Only 2 cases have been reported in the literature on vitiligo affecting the gingiva [1, 9]. Involvement can result in partial or total loss of skin pigmentation, often in irregular, sharply defined patches which may or may not be surrounded by macroscopic hyperpigmentation.

The precise etiology of vitiligo remains unknown. In the affected skin there is a lack of functional melanocytes. There are different hypotheses postulated regarding the pathogenesis of this condition of which the most important one is the “*autoimmune hypothesis*.” Autoimmune hypothesis states that antibodies produced by the body target melanocytes and destroy them. The second hypothesis is the “*neural hypothesis*,” which suggests that the altered reaction of melanocytes towards neuropeptides is responsible for melanocyte destruction. The third hypothesis is the “*self-destructive hypothesis*,” which states that melanocytes destroy themselves due to defects in protective mechanisms removing toxic melanin precursors. The last hypothesis is the “*biochemical hypothesis*,” which assumes that oversynthesis of hydriobiopterin, a cofactor of tyrosine hydroxylase, results in increased catecholamine synthesis [4].

Many authors have observed an increased frequency of autoimmune and autoinflammatory diseases particularly autoimmune thyroid disease (Graves' disease and autoimmune hypothyroidism), latent autoimmune diabetes in adults, rheumatoid arthritis, psoriasis, pernicious anemia, systemic lupus erythematosus, and Addison's disease in generalized vitiligo patients [10, 11]. Genetic analysis has identified several chromosomal regions which can contribute to both vitiligo and autoimmune disorders [12].

Vitiligo has been classified into three types, localized, generalized, and universal types. These are based on the distribution pattern. Localized type includes focal, mucosal, and segmental. The generalized type includes acrofacial, vulgaris, and mixed, while the universal is defined as complete or nearly complete depigmentation [7, 13].

A thorough clinical history along with clinical presentation and histological analysis is necessary for the correct diagnosis of vitiligo. Differential diagnosis for vitiligo includes nevus depigmentosus, lichen sclerosus, and chemical leukoderma. The most commonly encountered condition is nevus depigmentosus which is a congenital and stable leukoderma and has a discrete, regular, or serrated appearance, whereas vitiligo is acquired and characterized by patches devoid of melanocytes. Microscopic examination of lichen sclerosus is characterized by reduced thickness of epithelium accompanied by hydropic degeneration of basal cells. Unlike in vitiligo, active melanocytes can be identified in the lichen sclerosus epithelium. Chemical leukoderma is an acquired hypopigmentation caused by repeated exposure to certain chemicals containing phenolic group.

Here, the patient manifested with a localized vitiligo which was seen to be affecting only the gingiva. Complication arising due to vitiligo is mostly cosmetic, which can in turn affect the mental status of the patient [1]. Vitiligo lesions involving lips and oral mucosa are more resistant to medical therapies, as no melanocyte reservoir exists in these areas because of an absence of hair follicles. In early vitiligo, topical tacrolimus is seen to be effective to some extent. Topical pimecrolimus has been found to be effective in some cases of mucosal depigmentation [4]. Cosmetic correction of gingival vitiligo has been attempted by tattooing [1]. However in this case, the patient had no cosmetic concerns and did not want to undergo any treatment for the cosmetic debility.
